# Type 2 Innate Lymphoid Cells: Friends or Foes—Role in Airway Allergic Inflammation and Asthma

**DOI:** 10.1155/2012/130937

**Published:** 2012-11-11

**Authors:** Abbas Pishdadian, Abdol-Reza Varasteh, Mojtaba Sankian

**Affiliations:** ^1^Immunology Research Center, School of Medicine, Mashhad University of Medical Sciences, Mashhad, Iran; ^2^Immuno-Biochemistry Lab, Allergy Research Center, School of Medicine, Mashhad University of Medical Sciences, Mashhad, Iran

## Abstract

Innate-like lymphocytes (ILLs) and innate lymphoid cells (ILCs) are two newly characterized families of lymphocytes with limited and no rearranged antigen receptors, respectively. These soldiers provide a first line of defense against foreign insults by triggering a prompt innate immune response and bridging the gap of innate and adaptive immunity. Type 2 innate lymphoid cells (ILCs2) are newly identified members of the ILC family that play a key role in type 2 immune responses by prompt production of type 2 cytokines (especially IL-5 and IL-13) in response to antigen-induced IL-25/33 and by recruiting type 2 “immune franchise.” Regarding the two different roles of type 2 cytokines, helminth expulsion and type 2-related diseases, here we review the latest advances in ILC2 biology and examine the pivotal role of resident ILCs2 in allergen-specific airway inflammation and asthma.

## 1. Introduction

Currently, it is widely accepted that the innate immune system is not just a simple physical barrier to keep intruders out, but also a director of the immune system. This system not only recognizes and responds to foreign insults as an independent immune system, but also has an indisputable role in triggering, directing, and regulating adaptive immunity. To do so, it needs several cell types to be executive cells of innate systems. Hence, the study of these cells' biology, molecular mechanisms, and interactions has been an interesting area of research since their discovery. Identification of a new innate cell type, innate lymphoid cells, to immune system study had a great impact on our understanding of how the immune system works in physiologic and pathologic situations. In this paper we will review the biology of innate and innate-like cell families and examine type 2 innate lymphoid cells in details, focusing on their role in asthma and other airway inflammatory disorders.

## 2. Innate-Like Lymphocytes (ILLs)

Several cell types belong to the innate arm of the immune system. All these cells are originally myeloid/monocyte or lymphoid derived. Eosinophils, basophils, mast cells, neutrophils, monocytes, macrophages, and dendritic cells are myeloid/monocyte derivatives of innate cells. Members of this family have no antigen-specific receptors, which defines them as part of innate immunity, and some share both innate and adaptive immune characteristics. 

The other member of the innate cell family, lymphoid derived, can be divided into two groups: innate-like lymphocytes (ILLs) [1] and innate lymphoid cells (ILCs) [2].

Innate-like lymphocytes (ILLs) have rearranged antigen-specific receptors with limited diversity and tissue distribution. They are incapable of recognizing and responding to antigens as specifically and powerfully as adaptive cells. For this reason, they provide a first line of defense against foreign invaders. This rapid and innate-like response bridges a gap between innate and adaptive immunity. Members of this innate-like lymphocyte (ILL) family include mucosal-associated invariant T cells (MAITs) [3–6], invariant natural killer T cells (iNKTs) [7–10], *γδ* T cells (T*γ*/*δ*) [11–16], marginal zone B cells (MZB) [17–20], and B1-B cells [21–28]. The biology and function of the ILL family are summarized in Table 1. 

## 3. Innate Lymphoid Cells (ILCs)

The term “innate lymphoid cells” (ILCs) generally refers to an innate cell population with common phenotypic and functional features. Unlike adaptive immune cells, ILCs lack rearranged antigen-specific receptors and therefore are able to react promptly to a wide range of signals. They also have indisputable roles in tissue homeostasis, lymphoid tissue formation, and tissue repair. The ILC family may arise from a common progenitor, but members show three functionally distinct features. These three subsets include natural killer cells (cytotoxic ILCs) [29–35], ILCs with the retinoic acid (RA) receptor-related orphan receptor (RoR) *γ*t transcription factor (RoR*γ*t+ ILCs) [36–39], and type 2 ILCs (ILCs2) [2].

The biology and functions of the ILC family are summarized in Table 2. In this paper, we focus mainly on type 2 ILCs; therefore details about the other two members of ILC family are more complete than those of type 2 ILCs in Table 2.

## 4. Type 2 Innate Lymphoid Cells (ILCs2)

Type 2 innate lymphoid cells (ILCs2) consist of a heterogeneous cell population that shares phenotypic features and also depends on the common *γ* chain (*γ*c) of the IL-2 receptor and the transcription repressor Id2 [2]. These are divided into five categories (Table 3): natural helper cells (NHCs), nuocytes, type 2 innate helper cells (Ih2), type 2 innate lymphoid cells (ILCs2), and multi-potent progenitor population type 2 (MPP^type2^) cells.

### 4.1. Discovery and Naming

In 2001, a non-B, non-T, non-NK lymphoid cell type was described that released the type 2 cytokines IL-4, IL-5, and IL-13 in response to IL-25 (IL-17E) administration in mice [40]. These cells were RAG independent and *γ*-common chain dependent. A decade later, several research groups [42, 50, 53] simultaneously reported the same populations (Lin^−^ sca1^+^ Thy1^+^ T1/ST2^+^) but named them differently. All these cells, which are at the final stage of differentiation, produce IL-5 and IL-13 in response to IL-25 and IL-33 (IL-1-like cytokine). These cells were first identified in mice and named natural helper cells (NHCs), in view of their ability to help B1 cells produce antibody [42], nuocytes [50], in view of their IL-13 production (nu = the 13th letter of the Greek alphabet), and type 2 innate helper cells (Ih2) [53]. Nuocytes and Ih2 cells have similar surface markers, with the exception of Ih2 cells being Sca-1 negative, suggesting a close relationship between them. 

Similar cell populations with surface markers CD161 and CRTH2, a high affinity PGD2-R, were described in human, which were in their final differentiation state [55]. It is not clear whether these different reported cell types are truly the same or belong to distinct populations. These cells were recognized by IL-13- and IL-4-green fluorescent protein (GFP) reporter mice and selective-deficient mice. 

Another IL-25 responsive type 2 cytokine producer of the ILC2 family is able to differentiate into both monocyte/macrophage and myeloid lineages. These are termed multipotent progenitor population type 2 (MPP^type2^) cells [56]. All these cell types have been described in other studies [43–45, 51, 52]. These similar cell populations that are distinct from other innate cell types, especially in surface phenotype (CCR3^−^ CD49b^−^ FC*ε*RI*α* ^−^ Lin^−^), are categorized as new members of the innate lymphoid cell family. The biology and functions of the ILC2 family are summarized in Table 3.

### 4.2. Tissue Distribution

Natural helper cells (NHCs) were first observed in fat-associated lymphoid clusters (FALCs) of the intestinal mesentery, in fatty deposits in the peritoneal cavity, and surrounding the kidneys [42]. In another study, it was found that NHCs are also resident in lung tissue of mice [2]. Nuocytes were identified in mesenteric lymph nodes (mLNs), spleen, intestine, and in low abundance in peripheral blood [50]. Type 2 innate helper cells (Ih2) have a broad tissue distribution but are abundant in mLNs, liver, and spleen [53].

MPP^type2^ cells are mostly found in mLNs and gut-associated lymphoid tissues (GALTs), such as cecal and Peyer's patches [56]. Human type 2 innate lymphoid cells (ILCs2) are present in gut and lungs of human fetus and adult, palatine tonsils, and peripheral blood of human adults [55]. ILCs2 are more abundant in fetus (0.2% in lung and up to 2% in intestines) than adult (less than 0.1% of human CD45+ cells in tissues and 0.01–0.03% in peripheral blood) [55].

### 4.3. Developmental Origins and Effective Factors

The questions about the developmental origins of the ILC2 family are not fully answered but some progenitors have been proposed (reviewed in [43]): these are (1) the common lymphoid progenitor (CLP), which may have the potential to differentiate directly to the ILC2 cell type; (2) ILC2 cells, which may have their own distinct progenitor that may arise from CLPs; (3) MPP^type2^ cells, which possess the capacity to generate multiple lineages; myeloid/monocytic/lymphoid in two potent ways: MPP^type2^ cell is a homogeneous population in which each individual cell can generate multiple lineages, or MPP^type2^ cells are heterogeneous and consist of different progenitors [43]. Recent study revealed that nuocytes are derived from the CLP in bone marrow [52]. They could also result from a pro-T cell in the DN1/DN2 developmental stage [52]. These results indicate that ILC2 cells are more closely related to Tcells than any other immune cells. Fresh blood-separated human ILCs2 show more plasticity than tissue-derived ILCs2 [55]. Using RAG-1/ROSA26 (YFP) mice, Yang et al. indicated that NHCs were derived from bone marrow lymphoid progenitors [46]. In this study, lymphoid but not myeloid-erythroid progenitors were able to give rise to natural helper cells in vivo. The cytokine receptor Flt3, which is needed for the efficient generation of bone marrow lymphoid progenitors, is a key factor for NHC development [46]. There is little doubt regarding the derivation of ILC and ILC2 families from common lymphoid progenitors. As described in Section 3 and Table 2, ILC2 populations belong to the innate lymphoid cell (ILC) family along with two other members: NK cells and RoR*γ*t+ ILCs. They all share a phenotypic (lymphoid) homology and a common dependence on transcription repressor Id2 and the *γ*c chain of the IL-2 receptor [2]. It seems likely then that these three ILC family members derive from a common precursor distinct from the B cell/T cell precursor and that all of those precursors in turn derive from a common lymphoid progenitor. In other words, CLP may give rise to several distinct progenitors, one with a potential to change to ILC subsets and another with a potency to differentiate to ILC2 subsets. This has been supported by the finding that Id2 overexpression in hematopoietic precursors inhibits B cell, T cell, and plasmacytoid dendritic cell development while promoting ILC differentiation (reviewed in [57]). In fact, Id2, in association with transcription factor (TF) Tox, commits CLP to a common ILC progenitor that is capable of development towards type 2 ILCs and RoR*γ*t+ ILCs in the presence of ROR*α*/GATA-3 and ROR*γ*t/AhR, respectively [57].

MPP^type2^ cells are dependent on IL-3 and stem cell factor (SCF) for in vitro survival growth, and expansion [56]. SFC and IL-7 are required for survival, and IL-2, with or without IL-25, is necessary for proliferation in NHC cultures [42]. Survival, and expansion of nuocytes is IL-7 and IL-33 dependent [50]. All ILC2 family members activate and release IL-5 and IL-13 in response to IL-25, IL-33, and helminth infections [42, 50, 53, 55, 56]. NHC and nuocytes are more IL-7 dependent (for development and survival) than other ILC2 family members. Human ILCs2 can expand and produce type 2 cytokines in response to IL-2/IL-25 or IL-2/IL-33 combinations [55]. Transcription repressor Id2 has an important role in the development of all ILC2 family members [2]. This raises the possibility that one of the E proteins, which are Id2 blocker ligands, may arrest ILC2 development. The transcription factor RoR*γ*t, which has a pivotal role in RoR*γ*t+ ILCs development, has no definite role in mouse ILC2 generation but expressed in low abundance by human ILC2 [55]. Other transcription factors involved in ILC2 development are c-Maf, Jun-b, and T-bet for NHCs [42] and Gata-3 and STAT-6 for NHCs, nuocytes, and Ih2 cells [50, 53]. Ih2 cells are also aiolosdependent. Aiolos is a Th2 transcription factor [53]. It has recently been shown that nuocytes are dependent on Notch1 and RoR*α* [52]. MPP^type2^ cells express no or low transcripts of STAT-6, GATA-3, and c-Maf [56]. In a Doherty et al. study [47], mouse lung and bone marrow NHCs and human peripheral blood ILCs2 constitutively expressed ETS-1 transcription factor, in addition to GATA-3 and STAT-6. These transcription factor pathways (STAT-6, GATA-3, and ETS-1) may contribute to ILC2 proliferation, and Th2-type responses were seen in Alternaria-induced asthma [47]. 

### 4.4. Biologic Functions

It is now obvious that the most important role of ILCs2 is type 2 cytokine production, especially IL-5 and IL-13. The function of cell surface markers and other ILC2 molecules in cell-cell interactions is not fully understood.

#### 4.4.1. Crosstalk with Other Cell Types

ILCs2 are involved in cell-cell interactions with other hematopoietic cells. FALC resident NHCs can induce peritoneal B1-B cells to renew themselves and also splenic B cells to produce IgA [42]. Inducible costimulator (ICOS), an important factor in germinal center formation, is highly expressed on ILCs2, especially on nuocytes, where ILCs2 can interact with ICOS ligand (ICOSL) on B cells. However, the role of ILCs2 in B cell regulation has not yet been fully elucidated [50]. There may be a dialogue between ILCs2 and T cells, based on experimental studies. There is evidence that ILCs2 are able to trigger and promote Th2 cells, and in turn Th2 cells support ILCs2 [42]. Mice with a deficiency in IL-17RB, a part of IL-25R, show a reduction in frequency of both ILC2- and IL-13-producing T cells. ILCs2 adoptive transfer to such mice rehabilitates antigen-specific IL-13 production by T cells [42]. On the other hand, although the number of ILCs2 was increased in RAG^−/−^ mice infected with *Nippostrongylus brasiliensis*, they are not maintained. So ILCs2 are necessary to mount a Th2 response, and, in turn, Th2 cells support IL-5 and IL-13 production by ILCs2. Saenz et al. demonstrated that MPP^type2^ cells can present antigens and also promote Th2 responses [56]. Nuocytes express MHC-II, so they could hypothetically present antigens to T cells [50]. 

ILCs2 can also interact with nonhematopoietic cells. Human ILCs are abundant in fetal gut, even before microflora colonization. Although the function of ILCs has not been elucidated in gut formation and homeostasis, their presence suggests they may have a potential role [55]. ILCs constitutively express IL-13 transcripts, and this cytokine may promote fetal gut formation. In two separate studies, it has been shown that ILCs2 interact with lung cells and are involved in in lung tissue protection and repair [46, 57]. Influenza virus-induced airway hyperresponsiveness (AHR) was exaggerated in ILC2 depletion, as shown by Monticelli et al. [48]. ILCs2 can reduce AHR through production of amphiregulin, a member of the epidermal growth factor (EGF) family, because this substance can affect epithelial cell integrity, lung function, and airway remodeling [48]. Hence, the production of tissue-protective materials could be one the mechanisms of ILC2 involvement in lung tissue homeostasis. Production of these wound-healing molecules has also been demonstrated for alveolar macrophages [49].

#### 4.4.2. Immunity against Helminth Infections

Type 2 immune responses are important in defense against all helminth infections. The roles of IL-25 and IL-33 in immune responses against helminths have been demonstrated. ILCs2 are considered to be major IL-25 and IL-33 responders, so these cytokines stimulate ILCs2 to produce the type 2 cytokines IL-5 and IL-13 [2].

IL-13 induces production of resistin-like molecule b (RELMb), an antinematode protein [2]. IL-13 can also trigger the secretion of IgE [58]. IL-5 induces eosinophil differentiation and recruitment from bone marrow [58]. Taken together, the above studies suggest ILCs2 could be the executive arms of IL-25 and IL-33 on helminth immunity. This idea has been proved by the study through which transfer of purified in vitro-cultured ILCs2 to IL-25 and IL-33-deficient mice rescued them from *N. brasiliensis* infection. In experiments by Yasudaa et al., Il-33 activated ILCs2 populated in lungs and triggered pulmonary eosinophilia in *Strongyloides venezuelensis*-infected mice even in absence of adaptive immune cells [59]. 

In general, cooperation of ILCs2 and Th2 cells leads to recruitment and activation of other type2 “immune franchise” including eosinophils, basophils, mast cells, and IgE-producing B cells, as well as goblet cell hyperplasia, resulting in effective immune responses against helminths [2].

#### 4.4.3. Airway Pathology

In type 2 response-related disorders, type 2 immune cells and cytokines cause tissue damage and pathologic inflammatory conditions [60]. Airway inflammation and damage are the most relevant type 2 pathologies that result from airway allergy and asthma. The role of ILCs2 in type 2-related diseases can be considered from two points of view. (1) IL-25 and IL-33 are type 2 cytokines; their significant roles in type 2 airway allergy have been clearly demonstrated [61–65]. The study of their molecular mechanisms is an attractive area for basic and clinical research. ILCs2 as the major IL-25/IL-33 responsive cells could be considered as downstream effectors of these cytokines [54, 66]. (2) Regarding IL-5 and IL-13 as potent type 2 inducers, it is reasonable to propose important roles for ILCs2 in type 2 airway pathologies such as allergy and asthma [48, 52, 67–70].

In this section, we review the significant roles of IL-25 and IL-33 and their main responder cells, ILCs2, in airway allergic diseases and asthma.

IL-33 is a newly identified member of the IL-1 cytokine family [71]. It has a heterodimeric receptor, IL-33R, consisting of ST2 (also known as IL-1RL1, T1, DER4, IL-1R4, and Fit-1) and IL-1R accessory protein (IL-1RAcP) [71]. IL-33 is produced by a variety of cells and tissues including human and mouse lung tissue, lung stromal cells, airway epithelial cells, airway smooth muscle cells, and alveolar macrophages (reviewed in [61, 62]). IL-33-activated type 2 “immune franchise” produces a wide spectrum of type 2 cytokines (reviewed in [63]). Three independent tools have been used to disclose the role of IL-33 in promoting airway allergy and asthma [63]; these are (1) genetic studies: in the case of IL-33 and/or ST2 gene polymorphisms or genomewide association studies (GWAS), (2) evaluations of IL-33/ST2 cellular gene expression and intracellular signaling pathways in allergic and asthmatic mice, for example, by ST2 blockage or IL-33 inhibition, and (3) study of IL-33-defective mice, and also evaluation of IL-33 administration to such mice. Results of these studies demonstrated that IL-33 increases in clinical and experimental models of asthma so that its levels correlate with disease severity and that IL-33 and/or ST2 blockage reduce symptom severity [61–63]. IL-33 can induces anaphylactic shock when it is associated with IgE [61]. Another example of the significant role of IL-33 in allergic/asthmatic reactions is that IL-33 was overexpressed in skin cells of patients with atopic asthma, and degradation of IgE-primed skin mast cells was mediated by IL-33. IL-33 administration also induced AHR and goblet cell hyperplasia even in the absence of adaptive immunity [61]. It has been shown that IL-33 localizes to the nucleus and is probably released from damaged cells and tissues, because it is seen in allergen-mediated airway pathology. Hence, IL-33 may act as a nuclear alarm for innate immunity after damage to, or infection of, epithelial barriers [64]. Therefore, in a general view, the active role of IL-33 in triggering of type 2 immune responses has dual outcomes: (1) a protective one in the case of infections by promoting tissue repair and damage containment mechanisms and (2) a detrimental one in appreciation of type 2 immune-related diseases, marked airway allergic inflammation, and asthma [62]. 

IL-25 is a member of the IL-17 family (IL-17E) that binds to its heterodimeric receptor (IL-25R), IL-17RA/IL-17RB [40]. IL-25 is another newly identified type 2 cytokine that induces type 2-related disorders [40]. In a murine model of atopic dermatitis, dermal dendritic cell-derived IL-25 could demolish epithelial barrier function through both Th2-response induction and inhibition of keratinocytes to filaggrin, a necessary factor for skin barrier development [65]. 

Several studies indicated ILCs2 in allergic reactions and asthma as major responders to IL-25 and IL-33 and significant inducers of type 2 responses. In two studies [54, 67], virus- and glycolipid-induced AHR activated macrophages to produce IL-33, which in turn led to accumulation of ILCs2 and type2 cytokine-mediated exacerbation of inflammation. These studies showed that depletion of ILCs2 with anti-Thy1 antibodies significantly reduces virus-induced AHR. Furthermore, adoptive transfer of ILCs2 to IL-13^−/−^ mice restored AHR in both virus- and glycolipid-induced asthma. These studies show that ILCs2 can induce lung pathology even if they were the only source of IL-13. In the virus-induced AHR model by Chang et al., two functionally distinct ILCs2 were speculated to exist in the lung [67]: (1) ST2^+^ cell types; probably natural helper cells (NHCs) that produce IL-13 in response to IL-33 and contribute to virus-induced AHR. These cells have no impact on T-cells mediated allergen-induced asthma and therefore are not necessary for allergen-specific Th2 differentiation and (2) IL-17RB^+^ lung-resident cells, probably nuocytes, that trigger Th2 responses in a manner dependent on IL-33 and/or IL-25. Recently, Wolternik et al. have shown that ILCs2 are resident cells in lungs and mediastinal lymph nodes [68]. Intranasal administration of IL-33 and IL-25 caused an asthmatic phenotype in mice by increased accumulation of ILCs2 in lungs and bronchoalveolar fluids. Using IL-5 reporter mice, a non-T lymphoid cell type was recognized in the lung with similar phenotype and cytokine patterns, but not identical to ILCs2 [69]. These cells produce IL-5 and recruit and maintain eosinophils to the lungs in response to IL-25, and more effectively, to IL-33.

The link between ILCs2 and the adaptive immune system has been addressed. In OVA and house dust mite- (HDM-) induced experimental asthma, ILCs2 are the main source of IL-5 and IL-13 production. This research team [68] also revealed that ILC2 activation could occur even in absence of T cells in RAG^−/−^ mice. In support of this finding, Barlow et al. showed that ILCs2, and not Th2 cells, are the main actors of airway inflammations, in OVA-induced experimental asthma [70]. In OVA-induced asthma there was a similar number of Th2 cells and ILCs2 in lung, perhaps because of a need for T-cell-mediated specific recognition of OVA peptides. Interestingly, even in this asthmatic model, ILCs2 were the main source of IL-5, but both cell types produced equal amounts of IL-13 [68, 70]. In experimental ova- and protease-induced asthma models, Oboki et al. demonstrated that IL-33 is an amplifier of innate rather than acquired immune responses [72]. In a Hammad et al. study, HDM-induced asthma activated airway epithelial cells in an LPS-TLR4 interaction-dependent manner [73]. This activation in turn increased the production of IL-33, IL-25, and thymic stromal lymphopoietin (TSLP); raising the possibility that resident ILCs2 might be activated by these stroma-derived cytokines in HDM-induced asthma. Halim et al. showed a similar ILC2 activation in a protease allergen-induced asthma model [66]. Therefore, the role of ILCs2 in asthma seems to be as a primary translator of allergen-induced stroma-derived signals, which result in type 2 cytokine production and pathology by these cells. These massive stimulations subsequently will include the adaptive immune system as well. In conditions of T-cell activation, as in OVA-induced asthma, for example, ILCs2 seem to be downstream amplifiers of inflammation from T cells. This finding was confirmed by a recent study in which ILC2 activation by intranasal administration of papain, a protease allergen, temporarily mediated IL-9 production [74]. IL-9 production depended only on acquired immunity-derived IL-2, suggesting a functional link, and possible dependency, of these innate cells to the adaptive immune system. The dependency of ILCs2 to adaptive immunity both for cytokine production [74] and survival/maintenance (Section 4.3) could alter the conventional thinking that lack of adaptive immune cells can only affect adaptive sources of cytokine production. 

## 5. Concluding Remarks and Future Directions

ILCs2 are a heterogeneous subset of the ILC family that can be subdivided on the basis of surface phenotype and cytokine production patterns. The origin of ILCs2 has not been conclusively identified. It is not fully understood whether ILCs2 are truly different subsets with distinct development pathways or different responses of a single plastic cell type responding to environmental conditions and stimuli. Hence, they could be considered as members of the “type 2 franchise” that collectively mediate type 2-related airway pathology, such as allergy and asthma [60]. Whether ILCs2 can lead to this pathology alone or only following involvement of adaptive T cells remains to be fully understood. ILCs2, as a main source of type 2 cytokines, have a pivotal role in triggering other innate and acquired immune cells. Studies of ILCs2 by depletion or adoptive transfer have increased our understanding of the clinical manifestations of allergy and asthma. The need to understand ILC2 activators, signal transducing molecules, affected targets, and their mechanisms is urgent. In consideration of that, most of our knowledge about ILCs2 biology and function is based on in vitro and animal model studies. The question as to whether human ILCs2 are as important as animal ones in triggering type 2 responses and pathology remains to be answered and is an exciting area of future investigation.

## Figures and Tables

**Table 1:** Innate-like lymphocytes (ILLs) family.

Cell type	Date of discovery and naming in the first publish	Main development location	Major transcription factors and molecules needed for development	Anatomical locations	Developmental stage that derived from common progenitor	Major cytokines produced by cells	Major stimulating cytokines	Activator and proliferator cells	Antigen receptor	Ligands	Major surface markers	MHC/MHC-like restriction	Major functions and pathology	Antimicrobial defense mechanisms	Reference
MAIT	(i) Porcelli et al. 1993 [3] (ii) Tilloy et al. 1999 [4]	Thymus	PLZF (ZBTB16) in human	(i) Gut lamina propria (ii) Liver (human) (iii) Blood (few numbers)	Late double negative (DN)	IL-4 IL-5 IL-10 IL-17 IFN-*γ* TNF-*α*	IL-1*β*	(i) B cells (ii) Gut normal flora	TCR *α*/*β* V*α*19 (mouse) V*α*7.2 (human)	Hydrophobic molecules	IL-12R, IL-18R, IL-23R CD4^−^CD8^−^, CD8*α* ^+^ (human), CD161 (NKR-P1A), NKG2D, NKP30, P80, CCR6, CXCR6	MR1 (class Ib) restriction	(i) Possible regulation of inflammation in autoimmune disorders (ii) Regulation of IgA production in gut (iii) Intestinal homeostasis (iv) CNS pathology	(i) Cytokine production (ii) Cellular recruitment and recalling (iii) Cytotoxicity (Fas, granzymes)	[3–6]

iNKT	(i) In mouse: Koseki et al. 1990 [7] (ii) In human: Porcelli et al. 1993 [3]	Thymus	PLZF SLAM	Peripheral lymph tissues such as liver, spleen, and lymph nodes	Double positive (DP) (pro-T)	IL-4 IL-10 IL-13 IFN-*γ* TGF-*β*	IL-7 IL-12 IL-25 IL-33 TSLP	APCs	TCR *α*/*β* V*α*14 (mouse) V*α*24 (human)	Glycolipids especially *α*-Gal-Cer	DN or CD4^+^ (mouse), DN or CD8^+^ or CD4^+^ (human), CD28, ICOS, NK1.1, NKG2D	CD1d	(i) Immune regulation (ii) Immunity against infection (iii) Graft rejection and GVHD (iv) Pathogenesis of cancers, allergy, and autoimmune disorders	(i) Cytokine storm in a few minutes to hours (ii) Cellular recruitment and recalling	[3, 7–10]

*γ*/*δ* T cell	(i) Saito et al. 1984 [11] (ii) Samelson et al. 1985 [12]	Thymus	Sox13 Id3 Egr-1	(i) Epiderm (ii) Mucosal tissues (iii) Blood (few numbers)	DN2	IL-17 IFN-*γ* TNF-*α*	IL-2 IL-7 IL-21	(i) Mostly by antigens directly (ii) APCs (less common)	TCR *γ*/*δ*	Mostly nonpeptidic-self and nonself-antigens	NKRs: KIRs and NKG2A, C, D, TLRs, CCR7, CD16, CD226, MHC II, cytokine receptors	(i) Mostly no need to MHC restriction (direct recognition) (ii) MHC/MHC-like restriction (less common)	(i) Immune regulation (ii) Immunity against infection (iii) Tissue repair	(i) Cytokine production (ii) Cellular recruitment and recalling (iii) Cytotoxicity (Fas, granzymes, perforin)	[11–16]

MZB	MacLennan et al. 1982 [17]	Spleen marginal zone	BAFF Pyk-2 Notch2	(i) Spleen (ii) Blood (few numbers) (iii) Few numbers in Peyer's patches and lymph node subcapsular sinus	T1/T2 (immature B cell)	IL-6 IL-10 IFN-*β*	IL-7 IL-21	(i) APCs (ii) Mostly T independent (iii) Directly by antigen (iv) T dependent (less common)	BCR: IgM^hi^ IgD^low^	Mostly nonpeptidic antigens	CD1, CD9, CD27, CD36, CD11b, GP49b, TLRs, S1P1, MHC II, FC-like receptors, CD23^−^	CD1 (less common)	(i) Immunity against blood-borne infections (ii) Immunity against encapsulate bacteria (iii) Response to autoantigens derived from senescence	IgM and IgG3 production	[17–20]

B1-B cell	(i) Gronowicz and Coutinho 1975 [21] (ii) Hayakawa et al. 1983 [22]	Firstly in BM, then located in tissue	BAFF NFATc1 Siglec-G	(i) Peritoneal and pleural cavity (ii) Lungs (iii) Blood (few numbers)	T1/T2 (immature B cell)	IL-6 IL-10 TNF-*α*	IL-5	(i) Mostly T independent (ii) Directly by antigens	BCR: IgM^hi^ IgD^low^	Mostly nonpeptidic-self and nonself-antigens	CD1, CD5, CD19, CD20, CD22, CD27, CD9, CD11b, CD43, B220	CD1 (less common)	(i) Natural antibody production (ii) Immunity against infection (iii) Pathogenesis of autoimmune disorders and CLL	(i) IgM production (ii) IgG3 production (less common) (iii) IgA production in gut	[21–28]

MAIT: mucosal-associated invariant T cells, iNKT: invariant natural killer T cells, MZB: marginal zone B cells, MR1: MHC-related protein-1, Egr: early growth response, Id3: inhibitor of DNA binding-3, BAFF: B cell activation factor of the TNF family, Pyk: protein tyrosine kinase, Notch2: neurogenic locus notch homology protein 2, NFATc1: nuclear factor of activated T cell, cytoplasmic-1, S1P1: sphingosine-1-phosphate receptor-1, Sox: sex-determining region-(sry-) related high-mobility-group (HMG) box, PLZF: promyelocytic leukemia zinc finger, *α*-Gal-Cer: alpha-galactosylceramide, SLAM: signaling lymphocytic activation molecule, CLL: chronic lymphocytic leukemia.

**Table 2:** Innate lymphoid cells (ILCs) family^a,b^.

Cell type	Date of discovery and naming in the first publish	Anatomical locations	Major stimulating and development-effective cytokines	Major transcription factors and molecules needed for development	Major cytokines produced by cells	Major surface markers	Major functions and pathology	Reference
NK cells (cytotoxic ILCs)	(i) Kiessling et al. 1975 [29] (ii) Herberman et al. 1975 [30]	(i) Secondary lymphoid tissues (ii) Blood (iii) Mucosal tissues (iv) BM and thymus (less common)	(i) IL-12, IL-15, IL-18 (ii) IL-2, IL-21	Id2 Tox T-bet E4BP4 Eomes	IFN-*γ* IL-3 IL-10 TNF-*α* G-CSF GM-CSF CCL2 CCL3 CCL4 CCL5 XCL-1 IL-8	(i) Cytokine receptors^1^ (ii) Chemokine receptors^2^ (iii) Adhesion molecules^3^ (iv) Activating receptors^4^ (v) Inhibitory receptors^5^	(i) Innate responses against viral infections (ii) Immune surveillance against tumors	[29–35]

Ror*γ*t^+^ ILCs	First subset: Mebius et al. 1997 [36]	(i) Fetal lymphoid organs (ii) Tonsils, intestines, spleen, Peyer' patches	(i) IL-1*β*, IL-23 (ii) IL-7	Ror*γ*t Id2 Tox AhR Notch2 (just in adults)	IL-17 IL-22 IL-2 IL-13 GM-CSF TNF-*α* LT CXCL-18	(i) In all subsets: CD127 (IL-7R*α*), CD161, CD117 (c-kit), OX40L, CD30L (ii) In some subsets: CD4, CD90, CD56, NKP44 ( in human), NKP46 (in mouse), RANK, RANKL	(i) Lymphoid tissue formation (organogenesis) (ii) Tissue repair (iii) Innate immunity against bacterial and yeast infections (iv) Triggering of IgA production independently from T cells (v) Mucosal immunity^6^ (vi) Mucosal homeostasis^7^ (vii) Intestine and cancer pathology^8^	[2, 36–39]

Type 2 ILCs (ILCs2)	2001, 2002, 2010, 2011 (more detail in Table 3)	(i) FALC (ii) Lungs and gut (iii) mLN (iv) Palatine tonsils (v) Spleen (vi) Liver (viii) Peripheral blood	(i) IL-25, IL-33 (ii) IL-7	Id2	IL-5 IL-13	(i) In human: CRTH2 (GPR44), IL-7R*α*, IL-7RB, CD25, ST2 (ii) In mouse: CD44, Thy1, c-kit	(i) Helminth expulsion (ii) Tissue homeostasis (iii) Airway inflammation and remodeling	[2]

^a^The biology and functions of NK cells and Ror*γ*t^+^ ILCs are in more details than ILC 2. The latter cells are our target cells in this paper so we meet them in great details through the text. ^b^Each three major cell types of ILC family are actually a heterogeneous population so in this table just common features of subsets of each subfamily have been indicated.

^1^IL-1R, IL-2R, IL-12R, IL-15R, IL-18R, IFN-*α*. ^2^CCR2, CCR5, CCR7, CXCR1, CXCR3, CXCR4, CXCR6,CX3CR1, S1P5, Chem23R. ^3^CD2, CD56, CD122, DX5, CD11b, DNA M1, *β*1-integrins, *β*2-integrins. ^4^NKPs (NCRs), CD16, NKG2D, NKR-P1C, KIR-S, CD94/NKG2C, CRACC, ly9, CD84, NTB4, 2B4. ^5^LAIR1, KLRG1, NKR-P1B, NKR-P1D, TIGIT, CEACAM1, KIR-L, LILRB1, ly49, CD94/NKG2A.

^6^For example, by interaction with T cell, through CD30L and OX40L, that causes survival of Th2 and memory T cells. ^7^For example, by TNF-*α*-dependent activation of matrix metalloproteinases that lead to TGF-*β* production and in return IgA secretion by B cells. ^8^In intestine pathology, for example, by by IL-17 production and in return promotion and aggravation of mucosal inflammation. In cancer pathology, for example, by tumor expansion through recalling and recruitment immune suppressive cells.

Ror*γ*t: transcription factor retinoic acid (RA) receptor-related orphan receptor (Ror) *γ*t, Tox: thymocyte selection-associated high-mobility group box protein, Eomes: eomesodermin, T-bet (Tbx21): T-box transcription factor expressed in T cells, E4BP4 (NFIL3): nuclear factor IL-3 regulated (a basic leucine zipper transcription factor), AhR: aryl hydrocarbon receptor, LT: lymphotoxin, FALC: fat-associated lymphoid cluster, mLN: mesenteric lymph nodes, BM: bone marrow.

**Table 3:** Type2 innatel cells (ILCs2) populations.

Name	Date of discovery and naming in the first publish	Species	Tissue distribution	Cytokines produced by cells	Stimulating and development -effective cytokines	Transcription factors and molecules needed for development	Surface phenotype	Functions and pathology	Reference
Natural helper cells (NHCs)	(i) Fort et al. 2001 [40] (ii) Hurst et al. 2002 [41] (iii) Moro et al. 2010 [42]	Wild-type mouse	FALT Lung	*(i) IL-5, IL-13 (ii) IL-4, IL-6, IL-2 IFN-*γ*	*(i) IL-25, IL-33 (ii) IL-2/IL-25 (iii) SCF/IL-7 (iv) TSLP	*Id2, GATA-3, Stat-6, c-Maf, Jun-b, T-bet^low^, ETS-1	Lin^−^, c-kit (CD117), Sca1 (ly6a), IL-7R*α* (CD127), CD44, IL-2R*α* (CD25), ST2 (IL-33R), CD45, CD69, CD90.2 (Thy1.2), Flt3	(i) Nematode expulsion (ii) Induction of airway pathology following viral infection (iii) Tissue repair following influenza virus infection (iv) B1-B cell renewal (v) IgA production by splenic B cells (vi) Goblet cell hyperplasia	[40–49]

Nuocytes	(i) Neill et al. 2010 [50]	IL-13-GFP reporter mouse	Intestine mLN Spleen	*(i) IL-5, IL-13 (ii) IL-4, IL-6, IL-10 (iii) IL-2, GM-CSF (iv) CCL3 (MIP1*α*)	*(i) IL-25, IL-33 (ii) IL-7	*Id2, Notch-1, RoR-*α*, GATA-3, c-Maf, Stat-6, Jun-b	Lin^−^, c-kit^+/−^, CD44, MHC II, Sca1, IL-12RB, IL-7R*α* ^+/low^, IL-17RA, ICOS (CD278), Klrg1, ST2^+/−^, IL-10RB, IL-17RB (IL-25R), CD45, CD90.2, IL-27R (wsx1), ICAM-1, ICAM-2, CCR9, CXCR4, CXCR6, B7 integrin	(i) Nematode expulsion (ii) Increase in goblet cell mucin (iii) Increase in T-cell response	[43–45, 50–52]

Innate helper2 (Ih2) cells	(i) Price et al. 2010 [53]	IL-13-GFP and IL-4-GFP reporter mouse	Broad, mostly in lung, spleen, mLN, and liver	*(i) IL-5, IL-13 (ii) IL-4	*(i) IL-25, IL-33	*Id2, GATA-3, Stat-6, aiolos	Lin^−^, c-kit^+/−/low^, Sca1^−^, CD45, IL-2R*α*, CD90.2, CD44, ST2, CD69	(i) Nematode expulsion (ii) Increase in eosinophils	[43, 44, 52–54]

Type2 innate lymphoid cells (ILCs2)	(i) Mjösberg et al. 2011 [55]	Human	(i) Fetal/adult gut and lung (ii) Palatin tonsils (iii) Nasal polyps (iv) Adult peripheral blood	*(i) IL-5, IL-13	*(i) IL-25/IL-2 *(ii) IL-33/IL-2	*Id2, ETS-1, GATA-3	Lin^−^, c-kit^+/−^, IL-7R*α*, IL-2R*α*, ST2, IL-17RB, CD45, CRTH2 (GPR44, CD294) CD161 (NKR-P1A)	(i) Chronic rhinosinusitis	[47, 55]

Multipotent progenitor population 2 (MPP^type2^)	Saenz et al. 2010 [56]	IL-4-GFP reporter mouse	(i) mLN (ii) GALT especially in Peyer's patches and cecal patch	*(i) IL-5, IL-13 (mostly mRNA) (ii) IL-4 (mostly mRNA)	*(i) IL-25 (ii) SCF (iii) IL-3	*Id2, c-Maf^−/low^, GATA-3^−/low^ Stat-6^−/low ^	Lin^−^, c-kit, Sca1, CD45, ST2^−/low^, IL-17RB, CD34^−/low^, IL-7R*α* ^−/low^, CD62L^−/low^, **MHC II	(i) Nematode expulsion (ii) Increase in goblet cell mucin (iii) Increase in T-cell response	[43–45, 52, 56]

* The most important

** GFP^−^ cells expressed MHC II after cultivation with a combination of IL-3/SCF.

FALT: fat-associated lymphoid tissue, mLN: mesenteric lymph nodes, GFP: green fluorescent protein, GALT: gut-associated lymphatic tissues, Icos: inducible costimulatory molecule, CRTH2: chemoattractant receptor-homologous molecule expressed on Th2 lymphocytes, SCF: stem cell factor, GPR44: G protein-coupled receptor 44, ETS: E-twenty-six family, Flt3: Fms-related tyrosine kinase3.
